# The effect of Plan Do Check Act cycle management mode on patients with severe pneumonia complicated with respiratory failure: A retrospective study

**DOI:** 10.12669/pjms.41.8.12338

**Published:** 2025-08

**Authors:** Yinxian Shi, Yajuan Xu

**Affiliations:** 1 Yinxian Shi, Department of Nursing, Suzhou Hospital of Integrated Traditional Chinese and Western Medicine, Suzhou, Jiangsu Province 215101, P.R. China; 2 Yajuan Xu Department of ICU, Suzhou Hospital of Integrated Traditional Chinese and Western Medicine, Suzhou, Jiangsu Province 215101, P.R. China

**Keywords:** Blood gas, Inflammatory indicators, Plan-Do-Check-Act, Respiratory failure, Severe pneumonia

## Abstract

**Objective:**

To investigate the effects of Plan-Do-Check-Act (PDCA) cycle management nursing intervention on blood gas and biochemical indicators in patients with severe pneumonia complicated with respiratory failure (RF).

**Method:**

This retrospective cohort study was conducted at the Suzhou Integrated Traditional Chinese and Western Medicine Hospital and included records of 180 patients with severe pneumonia complicated with RF who were admitted for treatment between December 2022 to December 2024. Of them, 90 patients who received treatment based on the PDCA cycle management model (PDCA group) were matched in a 1:1 ratio with patients who received traditional nursing management (control group). Changes in blood gas, blood biochemistry, and self-care ability levels were compared between the two groups.

**Results:**

PDCA intervention was associated with significantly better levels of PaCO_2_, PaO_2_, and SaO_2_ compared to traditional nursing (P < 0.05). The average levels of procalcitonin (PCT), C-reactive protein (CRP), and interleukin-6 (IL-6) in the PDCA group were significantly lower than those in the control group (P < 0.05). The Evaluation of Self-Care Ability (ESCA) scores of the PDCA group were higher than those of the control group (P<0.05).

**Conclusion:**

Compared to routine care, PDCA-based nursing interventions can more effectively improve blood gas levels in patients with severe pneumonia complicated by respiratory failure (RF), reduce the inflammatory response, and enhance self-care abilities.

## INTRODUCTION

Severe pneumonia mainly results from the invasion of pathogenic microorganisms into lung tissue and often occurs in hospital respiratory departments.^1^,^2^ Patients with severe pneumonia are also prone to complications such as septic shock, multiple organ failure, and respiratory failure (RF),^3^,^4^ associated with very high (30% to 50%) mortality rates.^5^

In clinical practice, patients with pneumonia complicated by respiratory failure (RF) are often managed with continuous positive airway pressure ventilation, antibiotics, and electrolyte regulation.^6^ Although these treatment methods can improve the patient’s prognosis, studies have shown that high-quality care during the treatment process can further enhance the effectiveness of the treatment and improve the lung condition of RF patients.^7^ However, currently available targeted intervention measures in routine nursing are limited, making it difficult to achieve ideal intervention effects.^8^ The Plan-Do-Check-Act (PDCA) cycle management model is a scientifically based and effective quality management tool, also known as the “Deming Management Method.”^9^

The cycle consists of four stages: planning, implementation, inspection, and processing, where the endpoint of each stage serves as the starting point for the next stage.^10^ Recent studies have emphasized the effectiveness of PDCA-based nursing in managing critical care patients with complex respiratory conditions, including severe pneumonia and acute respiratory failure.^9^-^12^ Unlike previous studies that focused on general wards or single nursing outcomes, our study uniquely applies PDCA to patients with severe pneumonia complicated by respiratory failure (RF). Additionally, it comprehensively evaluates clinical effects through blood gas analysis, inflammatory markers, and self-care ability, offering a multidimensional perspective on patient outcomes.

## METHODS

This retrospective cohort analysis included 180 patients with severe pneumonia complicated by RF treated at Suzhou Traditional Chinese Medicine Hospital from December 2022 to December 2024. A cohort of 90 patients who received PDCA cycle management was matched with a cohort of patients who received traditional nursing management in a 1:1 ratio. Patients were matched manually in a 1:1 ratio based on key variables: age (±3 years), gender, BMI (±2 kg/m²), disease duration (±2 days), and lung lesion distribution (left, right, bilateral). Matching was performed manually using electronic medical records. All 180 eligible patients were successfully matched, resulting in a 100% matching rate. Propensity score matching (PSM) was not applied due to the limited sample size and partial missing data. However, future studies should consider using PSM to enhance comparability between groups.

### Ethical Approval:

The study was approved by the Institutional Ethics Committee of Suzhou Hospital of Integrated Traditional Chinese and Western Medicine (No.: 005; date: March 5, 2025), and written informed consent was obtained from all participants.

### Inclusion criteria:

- Meets the diagnostic criteria for severe pneumonia.^12^
- Meets the diagnostic criteria for RF.^13^
- Patients without other lung diseases such as pulmonary embolism, tuberculosis, bronchial asthma, etc.
- Complete clinical data.

### Exclusion criteria:

- Patients with concomitant malignant tumor diseases.
- RF caused by other reasons.
- Infection in other organs or systems.
- Patients with severe dysfunction of vital organs.
- Individuals with cognitive impairment, mental illness and poor treatment compliance.

### Nursing interventions:

Written informed consent was obtained from all patients before using structured questionnaires to collect social demography data and baseline characteristics.

### Traditional nursing:

After admission, patients received one-on-one routine education to enhance their disease-related knowledge. Their vital signs were closely monitored, and routine psychological counseling was provided. Routine care, such as sputum aspiration and oral care, was also provided.

### The PDCA cycle management mode:

Patients received nursing interventions based on the PDCA cycle management mode after admission, which comprised four steps.

***A) Step 1: Plan***. The nursing staff reviews the literature and analyzes relevant data from patients with severe pneumonia complicated by RF in the past, identifying factors and nursing issues that affect their efficacy, such as patient environment, compliance, diet, respiratory tract, and oral cavity. The individual nursing needs of each patient are assessed, and a nursing plan is developed.

***B) Step 2***, Do. Nursing staff provides care according to the established care plan that includes:

### Environmental care:

Regularly cleaning, ventilating, and disinfecting the ward; monitoring factors such as floating dust that may adversely affect patients’ breathing; reasonably controlling indoor temperature and humidity; ensuring a quiet and comfortable environment; alleviating negative emotions of patients; improve treatment compliance.

### Health education:

Providing health education to patients through oral, written, and visual means, including knowledge and information on disease causes, treatment methods, and preventive measures, to promote an accurate understanding.

### Psychological care:

Actively using a gentle tone to communicate with patients, actively listening to their demands, grasping their psychological status, and providing targeted psychological counseling; encouraging patients to alleviate their negative emotions and enhance confidence in treatment; encouraging family members to provide companionship and care, and enhancing their confidence in recovery.

### Condition observation:

Paying close attention to the patient’s condition development, monitoring heart rate, respiration, oxygen saturation, and other vital signs. In case of abnormalities, doctors are notified promptly for intervention. In cases where body temperature rises but does not exceed 38.5°C patients are promptly cooled by applying ice to the forehead, wiping the body with warm water, etc. When the temperature exceeds 38.5°C, the doctor is immediately notified, and the patient is administered antipyretic medication until the temperature returns to normal.

### Dietary guidance:

Developing a scientifically sound and realistic dietary plan for patients based on their individual dietary preferences and habits; Guiding patients to consume high-calorie, high-vitamin, light, easily digestible, and less irritating liquid foods, such as fish soup, egg soup, etc.; Supplementing vitamin A and iron appropriately to ensure balanced nutrition; Advising patients to consume more fresh vegetables and fruits to promote the discharge of toxins and phlegm. For critically ill patients, enteral nutrition solutions combined with nasal feeding are given according to medical advice. The patient’s head is raised appropriately within 30 minutes after nasal feeding to reduce the risk of aspiration.

### Respiratory and oral care:

Patient’s head is always kept tilted back to ensure airway patency; Patients are encouraged to drink around 1500mL of water daily to reduce the viscosity of sputum. If necessary, expectorants are administered, and attention is paid to eliminate foreign bodies in the respiratory tract. Patients are instructed to rinse their mouth after every meal. Paying close attention to the patient’s symptoms and guiding them to perform breathing exercises such as abdominal breathing and guiding the patient to actively cough to expel phlegm in cases of sputum accumulation. Methods such as tapping the back from bottom to top, from both sides of the back to the middle, or shaking the chest are used to further help the patient expel phlegm. When the accumulation of sputum is severe, nebulization inhalation or a sputum aspirator are used for sputum suction treatment.

*C) **Step 3 Check***. The implementation status of nursing work is evaluated weekly to ensure that nursing measures are fully implemented. Departments are organized to summarize and analyze nursing effectiveness, jointly discuss nursing-related problems, and propose solutions to develop more scientific and personalized nursing response measures.

*D**) Step 4 Act***. Organizing weekly symposia for patients, their families, and nursing staff to jointly discuss, summarize, and examine the results; incorporating unresolved issues into the next work plan, refining the nursing plan, and making arrangements for the next nursing stage. To enhance clarity, a summary of the key PDCA-based nursing actions has been provided in Supplementary Table-I.

**Supplementary Table-I:** Detailed Summary of PDCA-based Nursing Interventions.

Phase	Core Objectives & Focus	Specific Interventions
Plan	Comprehensive patient assessment and individualized care planning	Evaluate patient’s physical condition (respiratory function, nutrition), psychological state, treatment compliance, home care environment, and caregiver health literacy; develop targeted nursing plans accordingly
Do	Implementation of personalized nursing strategies	Deliver health education; provide psychological counseling; guide dietary intake; administer respiratory interventions (positioning, nebulized therapy, sputum management); perform oral care; reinforce nursing presence and patient engagement
Check	Monitoring of implementation efficacy and feedback collection	Conduct weekly reviews of nursing interventions; assess patient adherence, clinical response, and adverse events; organize interdisciplinary meetings to discuss care quality and outcomes
Act	Continuous improvement and optimization of care plans	Modify nursing strategies based on evaluation findings; reinforce ineffective measures; prepare revised care protocols for the next PDCA cycle

### Data collection:

PaCO_2_, PaO_2_, and SaO_2_ blood levels were measured using a blood gas analysis instrument (model ABL800 by Redo Company in Denmark).

Serum procalcitonin (PCT), C-reactive protein (CRP), and interleukin-6 (IL-6) levels were measured in 3ml of the patient’s fasting peripheral venous blood by chemiluminescent assay using the 7600 automatic biochemical analyzers (Hitachi, Japan). The reagent kit was purchased from Shanghai mblio Biotechnology Co., Ltd.

The self-care ability of patients was assessed using the Evaluation of Self-Care Ability (ESCA) scale that includes four dimensions: health knowledge level (19 items), sense of responsibility (six items), self-concept (seven items), and self-care skills (11 items), totaling 43 items. Each item is scored using a 0-4 point scoring method, with a maximum score of 172 points. The higher the score, the better the self-care ability. The ESCA scale has been validated in Chinese populations, showing high internal consistency (Cronbach’s α > 0.85) and good construct validity. It has been widely used in clinical nursing studies across China.^11^

### Data analysis:

Data were entered into Microsoft Excel and analyzed using SPSS version 20 (IBM Corp, Armonk, N.Y., USA). The Shapiro-Wilk test was used to evaluate the normality of continuous variables. Variables with normal distribution were expressed as mean ± standard deviation (SD) and compared using independent t-tests. Non-normally distributed variables were reported as median and interquartile range (IQR) and analyzed using Mann-Whitney U tests. Categorical variables were compared using chi-square tests. All p-values were two-sided, and p < 0.05 was considered statistically significant.

## RESULTS

A total of 180 patients (105 males and 75 females) were included in this study. The age range was 41-82 years, with a median age of 63 years (58-69 years). There were no significant differences in baseline data, including age, gender, BMI, disease course, and lung lesions, between the two groups. (P>0.05) (Table-I).

**Table-I:** Comparison of baseline data between two groups.

Daseline data	Control group (n=90)	PDCA group (n=90)	χ^2^/t/Z	p
Male (yes), n(%)	48 (53.3)	57 (63.3)	1.851	0.174
Age (years), mean±SD	64.9±8.2	62.9±7.8	1.675	0.096
Course of disease (day), M(P25/P75)	5 (4-6)	5 (3-5)	-1.530	0.126
BMI (kg/m^2^), mean±SD	23.8±2.7	23.1±2.8	1.700	0.091
***Lung lesions, n(%)***	32 (35.6)	36 (40.0)	2.911	0.233
Left lung	2.911	0.233		
Right lung	45 (50.0)	48(53.3)		
Bipulmonary	13 (14.4)	6 (6.7)		

Before the intervention, no significant differences were observed in PaCO_2_, PaO_2_, or SaO_2_ between the two groups (P > 0.05). After the intervention, both groups showed improvement, but the PDCA group demonstrated significantly better blood gas parameters compared to the control group (P < 0.05) (Table-II). Similarly, no significant differences were observed in baseline PCT, CRP, and IL-6 levels between the two groups. Following intervention, all three markers decreased significantly in both groups, with greater reductions noted in the PDCA group (P < 0.05) (Table-III). There were no significant differences in ESCA scores between the two groups before the intervention. After intervention, scores improved in both groups, with the PDCA group showing significantly higher scores across all four dimensions (P < 0.05) (Table-IV).

**Table-II:** Comparison of blood gas analysis between two groups of patients.

Index	Control group (n=90)	PDCA group (n=90)	t/Z	P
***Before intervention***				
PaCO2 (mmHg), mean±SD	47.6±6.0	48.4±6.9	-0.921	0.358
PaO2 (mmHg), mean±SD	78.7±6.7	77.9±7.4	0.688	0.492
SaO2 (%)	82 (77-90)	84 (78-90)	-0.562	0.574
***After intervention***				
PaCO2 (mmHg), mean±SD	72.8±6.6*	81.0±6.9*	-8.049	<0.001
PaO2 (mmHg), mean±SD	65.8±6.3*	49.4±6.2*	17.462	<0.001
SaO2 (%)	90.5 (84-95)*	93 (89-96)*	-2.667	0.008

***Note:***

* represents a comparison within this group compared to before care, *P*<0.05.

**Table-III:** Comparison of inflammatory response indicators between two groups, mean±SD.

Index	Control group (n=90)	PDCA group (n=90)	t	P
***Before intervention***				
PCT (μg/L)	2.91±0.46	3.00±0.41	-1.370	0.173
CRP (mg/L)	25.3±3.7	24.3±4.0	1.724	0.087
IL-6 (ng/L)	72.1±5.9	71.3±7.0	0.883	0.379
***After intervention***				
PCT (μg/L)	2.70±0.52*	2.39±0.43*	4.298	<0.001
CRP (mg/L)	18.7±3.2*	15.9±3.4*	5.636	<0.001
IL-6 (ng/L)	62.4±6.1*	57.5±5.2*	5.860	<0.001

***Note:***

* represents a comparison within this group compared to before care, *P*<0.05.

**Table-IV:** Comparison of self-care ability between two groups.

Index	Control group (n=90)	PDCA group (n=90)	Z	P
***Before intervention***				
Health knowledge level	38 (34-42)	36 (35-41)	-1.198	0.231
Self-responsibility	15 (12-16)	15 (13-17)	-1.155	0.248
Self-conception	19 (18-22)	19 (17-21)	-1.451	0.147
Self-care skills	20 (18-23)	21 (19-24)	-1.039	0.299
***After intervention***				
Health knowledge level	59.5 (58-64)*	69 (67-72)*	-10.022	<0.001
Self-responsibility	18.5 (16-20)*	23 (21-24)*	-8.439	<0.001
Self-conception	23 (21-25)*	27 (24-28)*	-6.665	<0.001
Self-care skills	32 (29-34)*	41 (39-43)*	-10.61	<0.001

***Note:***

* represents a comparison within this group compared to before care, *P*<0.05.

## DISCUSSION

This study aimed to explore the benefits of the PDCA management model in caring for patients with severe pneumonia complicated with RF. The results show that nursing intervention based on the PDCA circular management model markedly improved blood gas indicators and inflammatory markers compared to the traditional nursing method.

The observed improvements in arterial blood gas parameters in the PDCA group can be attributed to several pathophysiological mechanisms. First, the PDCA model emphasized enhanced respiratory tract care, including timely sputum clearance, chest physiotherapy, and nebulization, which alleviated airway obstruction and promoted alveolar ventilation. Second, better patient education and psychological support increased treatment compliance and reduced stress-induced respiratory burden. Third, environmental control and individualized care plans contributed to optimized ventilation-perfusion matching. Together, these factors may have led to improved oxygenation (PaO_2_ SaO_2_) and carbon dioxide elimination (PaCO_2_). These findings are consistent with prior literature on comprehensive respiratory care interventions.^14^

Our findings are consistent with recent PDCA-based clinical studies conducted in critical care settings, confirming improvements in patient outcomes and management quality.^14^,^15^ Compared to conventional nursing, nursing based on the PDCA cycle management model has clearer goals and targeted plans.^15^ During the initial planning stage, nursing staff analyze relevant historical information and develop targeted nursing plans tailored to the patient’s actual situation. This enables a planned and targeted approach to ensure that nursing intervention content is fully tailored to the patient’s exact needs.^16^ Providing personalized health education to patients can increase their health knowledge, effectively enhance their understanding of self-care skills, and thus improve their self-care ability. In addition, providing targeted psychological counseling to patients and introducing successful treatment cases can help maintain a positive mentality during the treatment period, improve patient confidence and treatment compliance, enable them to actively cooperate with nursing, and further contribute to improving their self-care abilities.^17^

Patients with severe pneumonia and RF have increased respiratory secretions due to lung infection, leading to pulmonary congestion and changes in arterial blood gas indicators.^1^,^2^,^18^ Studies have showed that a good and clean environment improves patients’ breathing conditions.^19^ In nursing interventions based on the PDCA cycle management model, effective improvement measures are implemented to address problems that occurred in the previous cycle, thereby avoiding repeated errors in the next cycle and making the subsequent nursing process more efficient, organized, and detailed.^19^,^20^ The check and act stages of PDCA further ensure the effectiveness of each nursing measure through weekly evaluation of nursing work implementation, organizing symposia and refining and perfecting nursing plans based on the evaluation results.^14^

Providing dietary care to patients, guiding them to consume nutritious foods, and providing enteral nutrition support can enhance the immune system and effectively reduce the inflammatory response.^17^,^18^ The implementation of the PDCA cycle management mode, therefore, transforms the nursing management process from focusing on previous management results to encompassing management factors, methods, and results, forming an integrated management mode that combines management, control, and monitoring. This, in turn, ensures the standardization and systematization of nursing plans.^21^–^24^ Timely observation of changes in the patient’s condition, targeted strengthening of nursing interventions, and providing patients with higher-quality and more meticulous care can all help reduce infection and inflammation. This study contributes to the nursing literature by applying the PDCA cycle model to a specific high-risk patient group—those with severe pneumonia complicated by respiratory failure—and demonstrating its effectiveness across clinical, biochemical, and behavioral domains. The findings underscore the clinical relevance of structured nursing interventions in improving oxygenation, reducing inflammation, and enhancing self-care ability. Strengths of this study include the matched cohort design, multidimensional outcome measures, and detailed intervention protocol. Future studies should focus on prospective validation and exploration of scalability in multi-institutional settings.

### Limitations

This study has several limitations. First, the retrospective design may introduce selection bias and limit causal inferences. Second, as a single-center study, the findings may not be generalizable to other clinical settings. Third, the relatively small sample size may reduce the statistical power to detect smaller effect sizes. Fourth, we did not employ advanced bias-reduction methods, such as propensity score matching. Future research should aim for a multicenter, prospective design with larger sample sizes and enhanced methodological rigor to validate these findings.

## CONCLUSION

Compared to routine care, the PDCA-based cycle management mode used to treat patients with severe pneumonia complicated with RF effectively improves blood gas indicators and achieves a better control effect on inflammation. The PDCA-based cycle management mode can also improve the patients’ self-care abilities. As such the PDCA-based cycle management approach shows potential for improving clinical outcomes in patients with severe pneumonia and respiratory failure, but further validation in multicenter, prospective studies is required before broad clinical implementation.

### Authors’ contributions:

**YS:** Study design, Literature search and manuscript writing.

**YS and YX:** Data collection, data analysis and interpretation. Critical Review.

**ZH:** Manuscript revision and validation and is responsible for the integrity of the study. All authors have read and approved the final manuscript.
